# Supplementary material for: Floquet Chern insulators of light
Source: Nat Commun. 2019 Sep 13;10:4194. doi: 10.1038/s41467-019-12231-4 (PMC6744472; doi:10.1038/s41467-019-12231-4)
Supplement: Supplementary file 1 — Supplementary Information [file 41467_2019_12231_MOESM1_ESM.pdf]

Supplementary Information
Floquet Chern Insulators of Light

He et al.

Supplementary Note 1: Solving the Floquet eigenvalue problem by modal decomposition

In this section, we present our general formalism to solve the Floquet eigenvalue problem of a nonlinear photonic crystal driven by an external monochromatic field. According to the Floquet theorem, the trial solution for electromagnetic fields propagating in the photonic crystal can be written in the form $\Psi_{\mathbf{k}}(t) = e^{i\mathbf{k}\cdot\mathbf{r}-i\varepsilon t}\phi(t)$, where $\phi(t)$ is a periodic function in both space and time $\phi(t) = \phi(t+T)$ and T is periodicity. Substitution into Maxwell equations yields the eigenvalue problem:

$$[A_{\mathbf{k}} - i\partial_t B(t)]\phi(t) = \varepsilon B(t)\phi(t) \tag{1}$$

where $A_{\mathbf{k}} = e^{-i\mathbf{k}\cdot\mathbf{r}}Ae^{i\mathbf{k}\cdot\mathbf{r}}$, $B(t) = B_0 + B_{\mathrm{nl}}(t) = B_0 + Ve^{-i\Omega t} + V^{\dagger}e^{i\Omega t}$. Our method is based by decomposing the time periodic function $\phi(t)$ using the eigenstates of the static eigenvalue problem without external drive ($|j\rangle$) as $\phi(t) = \sum_{jm} c_{jm}\,|j\rangle\, e^{im\Omega} = \sum_{jm} c_{jm}\,|jm\rangle$ and computing the coefficients c_{jm}. To this end, we substitute the expression of $\phi(t)$ back into (1) and multiply $\langle ln|$ from the left

$$\sum_{jm} c_{jm}\,\langle ln|\,[A_{\mathbf{k}} - i\partial_t B(t)]\,|jm\rangle = \varepsilon \sum_{jm} c_{jm}\,\langle ln|\,B(t)\,|jm\rangle \tag{2}$$

Using the orthonomal condition $\langle l|\,B_0\,|j\rangle = \delta_{lj}$ and $\langle n|m\rangle = \delta_{nm}$, we obtain

$$\begin{aligned}&\sum_{jm} c_{jm}[(\omega_j + n\Omega)\delta_{lj}\delta_{nm} + n\Omega V_{lj}\delta_{n,m-1} + n\Omega V^{\dagger}_{lj}\delta_{n,m+1}]\\ &= \varepsilon\sum_{jm} c_{jm}(\delta_{lj}\delta_{nm} + V_{lj}\delta_{n,m-1} + V^{\dagger}_{lj}\delta_{n,m+1})\end{aligned} \tag{3}$$

where $V_{lj} = \langle l|\,V\,|j\rangle$ and $\omega_j = \langle j|\,A_{\mathbf{k}}\,|j\rangle$. Furthermore, the above equation can be casted into a matrix form

$$\hat{M}\Psi = \varepsilon\hat{N}\Psi \tag{4a}$$

$$\Psi = (\dots, c_{j,-1}, c_{j,0}, c_{j,1}, \dots)^{\mathrm{T}} \tag{4b}$$

$$\hat{M} = \begin{pmatrix} \ddots & \ddots & & & & \\ -\Omega\hat{V}^\dagger & \hat{H}_0 - \Omega & -\Omega\hat{V} & & & \\ & 0 & \hat{H}_0 & 0 & & \\ & & \Omega\hat{V}^\dagger & \hat{H}_0 + \Omega & \Omega\hat{V} & \\ & & & \ddots & \ddots & \end{pmatrix}, \hat{N} = \begin{pmatrix} \ddots & \ddots & & & & \\ \hat{V}^\dagger & \hat{I} & \hat{V} & & & \\ & \hat{V}^\dagger & \hat{I} & \hat{V} & & \\ & & \hat{V}^\dagger & \hat{I} & \hat{V} & \\ & & & \ddots & \ddots & \end{pmatrix} \tag{4c}$$

with $(\hat{H}_0)_{lj} = \omega_j \delta_{lj}$. An equivalent way of writing (4a) is $\hat{N}^{-1}\hat{M}\Psi = \varepsilon\Psi$. As we expected, the matrix $\hat{M}$ on the left side of (4a) is non-Hermitian. In practice, to diagonalize the Floquet Hamiltonian, one can truncate the Fourier harmonics to a finite order ($|m| < m_c$), as the eigenstates $\{c_{jm}\}^{\mathrm{T}}$ are localized in m[1]. We set $m_c = 5$ in the calculations of both bulk and edge states dispersions. To prove the convergence, we first show c_{jm} is highly localized in m for a specific Floquet state (the bottom band at the Γ point in Fig.3d of the main text). As shown in Fig.1a, the two dominant components of c_{jm} indicate that the Floquet mode arises from the hybridization between Floquet basis $|1, m = -1\rangle$ and $|2, m = 0\rangle$, while the contributions from other Fourier orders are negligible. To further quantify the convergence of c_{jm} throughout all Floquet bands, we evaluate the worst-case scenario, defined as:

$$d(n) = \min\left(\sum_{|m|\leq n} \sum_{j=1,2} |c_{jm}(\mathbf{k})|^2\right) \text{ for } \mathbf{k} \in \text{B.Z.} \tag{5}$$

for the two Floquet bands in Fig.3d of the main text, respectively. The calculated $d(n)$ approaches 1 for $n \leq 2$ (Fig.1b), suggests our truncation of the Floquet basis at $m_c = 5$ is good enough for the calculation.

Supplementary Note 2: Proof of periodic spectrum

In this section, we prove the Floquet spectrum defined through (1) is necessarily periodic at the spacing of Ω. Specifically, if $\Psi_{\mathbf{k}}(t) = e^{i\mathbf{k}\cdot\mathbf{r}-i\varepsilon t}\phi(t)$ is a solution of (1) with eigenvalue ε and eigenstate $\phi(t)$, it is evident that $\Psi_{\mathbf{k}}(t) = e^{i\mathbf{k}\cdot\mathbf{r}-i(\varepsilon+\Omega)t}e^{i\Omega t}\phi(t)$ is also a solution with new eigenvalue $\tilde{\varepsilon} = \varepsilon + \Omega$ and eigenstate $\tilde{\phi}(t) = e^{i\Omega t}\phi(t) = \sum_{jm} c_{jm}\left|j, m+1\right\rangle = \sum_{jm} \tilde{c}_{jm}\left|j, m\right\rangle$, where $\tilde{c}_{jm} = c_{j,m-1}$. Compared to (4a), it implies shifting the column vector $\{c_{jm}\}^{\mathrm{T}}$ down (up) by one row also yields an eigenstate with eigenvalue increased (decreased) by Ω, though they represent the same physical state. Therefore, the resulting Floquet spectrum is periodic with a spacing of Ω.

Supplementary Note 3: Two-band simplification

In this section, we focus on a situation where only two of the bands are close to each other under the driving field ,while both bands are far away from the rest. Under rotating wave approximation, (4a) can be simplified into a series of 2-by-2 matrices by considering pairs of nearly-degenerate Floquet modes (e.g. ω_1 and $\omega_2 - \Omega$) and their coupling terms. As shown above, all these 2-by-2 matrices share the same Floquet eigenvalues, modulus Ω. Specifically, we can focus on one of them that reads:

$$\begin{pmatrix} \omega_2 - \Omega & -\Omega V_{21} \\ 0 & \omega_1 \end{pmatrix} \begin{pmatrix} c_{2,-1} \\ c_{1,0} \end{pmatrix} = \varepsilon \begin{pmatrix} 1 & V_{21} \\ V_{21}^{\star} & 1 \end{pmatrix} \begin{pmatrix} c_{2,-1} \\ c_{1,0} \end{pmatrix} \tag{6}$$

An equivalent way of writing the eigenvalue problem (6) is:

$$\frac{1}{1-|V_{21}|^2}\begin{pmatrix} \omega_2-\Omega & -(\Omega+\omega_1)V_{21} \\ (\Omega-\omega_2)V_{21}^\star & \omega_1+\Omega|V_{21}|^2 \end{pmatrix}\begin{pmatrix} c_{2,-1} \\ c_{1,0} \end{pmatrix} = \varepsilon \begin{pmatrix} c_{2,-1} \\ c_{1,0} \end{pmatrix} \tag{7}$$

It is evident that the two bands are decoupled as long as $V_{21}=0$ and the corresponding eigenvalues are $\varepsilon_{1,2}=\omega_2-\Omega,\omega_1$, respectively. In contrast, when $V_{21}\neq 0$, the new eigenvalues are

$$\varepsilon_{1,2}=\frac{|V_{21}|^2\Omega+\omega_1+\omega_2-\Omega\pm\sqrt{(|V_{21}|^2\Omega+\omega_1+\omega_2-\Omega)^2-4(1-|V_{21}|^2)\omega_1(\omega_2-\Omega)}}{2(1-|V_{21}|^2)} \tag{8}$$

For on-resonance driving ($\Omega=\omega_2-\omega_1$), the splitting of the two states is

$$\delta\varepsilon\approx 2|V_{21}|\sqrt{\omega_1\omega_2} \tag{9}$$

This is how we derived Eq. 2 in the main text. As shown, this eigenvalue problem is necessarily non-Hermitian but has real eigenvalues when $\omega_1\omega_2>0$.

Supplementary Note 4: Floquet photonic systems with and without time-reversal symmetry

In this section, we illustrate the conditions on preserving or breaking time-reversal (T) symmetry in a monochromatically driven nonlinear photonic crystal. Mathematically, T-symmetric means the matrix operator in the Floquet eigenvalue problem (4a) have the property of:

$$T(\hat{N}^{-1}\hat{M})_{\mathbf{k}}T^{-1}=(\hat{N}^{-1}\hat{M})^\star_{-\mathbf{k}} \tag{10}$$

When we look at individual terms of the matrices, it is easy to show that $T-$symmetry is equivalent to requiring:

$$V_{ij}(\mathbf{k})=V_{ij}^\star(-\mathbf{k}), \tag{11}$$

where the coupling terms are defined as:

$$V_{ij}(\mathbf{k}) = \langle i, \mathbf{k}| V |j, \mathbf{k}\rangle . \tag{12}$$

Here i, j label the bands and $\bar{\bar{\epsilon}}_{\rm nl}(t) = Ve^{-i\Omega t} + V^{\dagger}e^{i\Omega t}$ represents the effective permittivity tensor induced by the second-order optical nonlinearity of a material driven by an external electric field. As we started from a $T-$symmetric system in the static case, we have $|i, \mathbf{k}\rangle = |i, -\mathbf{k}\rangle^{\star}$. As a result, T can be broken in a second-order nonlinear optical material, when the coupling operator V is a complex tensor ($V \neq V^{\star}$). This can be achieved by driving the material with an elliptically polarized field. On the other hand, a linearly polarized field always lead to a real coupling operator, and the system is thus always T-symmetric.

Supplementary Note 5: Temporal analysis of the principle optical axes

Aside from the mathematical description of T-symmetry above, in this section, we provide an intuitive understanding on why some driving fields break T while others preserve T by analyzing the temporal evolution of the principle optical axes under a monochromatic driving field in an example material of $LiNbO_3$. Without driving field, $LiNbO_3$ is an uni-axial medium[2], where the principle optical axes - along which the permittivity tensor is diagonalzed - can be chosen as $(\hat{\mathbf{x}}, \hat{\mathbf{y}}, \hat{\mathbf{z}})$. In this coordinate, the permittivity tensor reads:

$$\bar{\bar{\epsilon}} = \begin{pmatrix} \epsilon_{xx} & 0 & 0 \\ 0 & \epsilon_{xx} & 0 \\ 0 & 0 & \epsilon_{zz} \end{pmatrix} . \tag{13}$$

Next we apply two specific examples of linearly and elliptically polarized drive, (1) x-polarized

and (2) circularly polarized, and analyze the evolution of instantaneous optical axes. Under an x-polarized driving field, $E_x^{\mathrm{d}} = E\cos\Omega t$, the only active second-order optical nonlinearity of $\mathrm{LiNbO_3}$ is $\chi^{(2)}_{zxx}$ (d_{31}), which gives rise to two terms in the effective permittivity, ϵ_{xz} and ϵ_{zx}:

$$\bar{\bar{\epsilon}}_{\mathrm{eff}} = \begin{pmatrix} \epsilon_{xx} & 0 & \alpha\cos\Omega t \\ 0 & \epsilon_{xx} & 0 \\ \alpha\cos\Omega t & 0 & \epsilon_{zz} \end{pmatrix}, \tag{14}$$

where $\alpha = d_{31}E$. Limited by the material damage threshold[3], the induced nonlinear permittivity change is always small: $\beta = \alpha/(\epsilon_{xx} - \epsilon_{zz}) \ll 1$. As a result, diagonalizing the effective permittivity tensor in (14) yields the new optical principle axes in the driven medium:

$$\hat{\mathbf{e}}_1 = (0, 1, 0)^{\mathrm{T}} \tag{15}$$

with $\epsilon_1 = \epsilon_{xx}$;

$$\hat{\mathbf{e}}_2 = (1, 0, \beta\cos\Omega t)^{\mathrm{T}} \tag{16}$$

with $\epsilon_2 = \epsilon_{xx} + \alpha\beta\cos^2\Omega t$; and

$$\hat{\mathbf{e}}_3 = (-\beta\cos\Omega t, 0, 1)^{\mathrm{T}} \tag{17}$$

with $\epsilon_3 = \epsilon_{zz} - \alpha\beta\cos^2\Omega t$. In such cases, one optical axis of $\mathrm{LiNbO_3}$ remains static $\hat{\mathbf{e}}_1 = \hat{\mathbf{y}}$; while the other two axes ($\hat{\mathbf{e}}_{2,3}$) are oscillating about z and x axes, respectively. Note these oscillations are T-preserving, which is easy to see as the substitution from t to $-t$ leaves all results unchanged.

On the other hand, under a circularly polarized driving field: $E_x^{\mathrm{d}} = E\cos\Omega t$ and $E_y^{\mathrm{d}} = E\sin\Omega t$, both $\chi^{(2)}_{zxx} = d_{31}$ and $\chi^{(2)}_{zyy} = d_{32}$ terms of $\mathrm{LiNbO_3}$ are active, and the new effective

permittivity tensor reads:

$$\bar{\bar{\epsilon}}_{\text{eff}} = \begin{pmatrix} \epsilon_{xx} & 0 & \alpha \cos \Omega t \\ 0 & \epsilon_{xx} & \alpha \sin \Omega t \\ \alpha \cos \Omega t & \alpha \sin \Omega t & \epsilon_{zz}, \end{pmatrix} \tag{18}$$

where $\alpha = d_{31}E = d_{32}E$. Again limited by the material damage, $\beta = \alpha/(\epsilon_{xx} - \epsilon_{zz}) \ll 1$, and the new optical principle axes become:

$$\hat{\mathbf{e}}_1 = (-\sin \Omega t, \cos \Omega t, 0)^{\text{T}} \tag{19}$$

with $\epsilon_1 = \epsilon_{xx}$;

$$\hat{\mathbf{e}}_2 = (\cos \Omega t, \sin \Omega t, \beta)^{\text{T}} \approx (\cos \Omega t, \sin \Omega t, 0)^{\text{T}} \tag{20}$$

with $\epsilon_2 = \epsilon_{xx} + \alpha\beta$; and

$$\hat{\mathbf{e}}_3 \approx (-\beta \cos \Omega t, -\beta \sin \Omega t, 1)^{\text{T}} \tag{21}$$

with $\epsilon_3 = \epsilon_{zz} - \alpha\beta$. As shown, all three optical axes rotate around the z-axis at the driving frequency Ω, and it is exactly this spinning behavior that breaks T-symmetry.

Supplementary Note 6: Berry curvature and Chern number calculations for Floquet bands

In this section, we define the Berry curvature for the Floquet bands, and explain how Chern number is defined. First, we reduce the Floquet eigenvalue problem at each $\mathbf{k}$ point into a non-Hermitian matrix eigenvalue problem as shown in Supplementary Note 1. We define the Berry curvature solely based on the right eigenstates of this eigenvalue problem as the Chern number defined in this ”Hermitian” way is the same as if we were to define it using both the left and right

eigenstates[4]. Specifically, we define Berry connection as:

$$\mathcal{A}_{\mathbf{k}} = -i \langle \Psi | \nabla_{\mathbf{k}} | \Psi \rangle \tag{22}$$

where Ψ are the column vectors that solve the matrix eigenvalue problem of (4a). Though this definition only uses the coefficients c_{jm}, the Chern number remains the same if we use the actual Floquet mode profiles of $c_{jm} |j, m\rangle$ as one can show the contributions from the cross terms $\langle j'm'| \nabla_{\mathbf{k}} |B_0 jm\rangle$ always add up to 0. Finally, we define Berry curvature as:

$$\mathcal{B}_{\mathbf{k}} = \nabla_{\mathbf{k}} \times \mathcal{A}_{\mathbf{k}}, \tag{23}$$

and define Chern number as the integral of $\mathcal{B}_{\mathbf{k}}$ over the entire Brilluion zone.

Next, we show two examples of the calculated Berry curvature of the Floquet bands. First we calculate for the T-symmetric example shown the previous section; specifically, we set $\Omega a/2\pi c = 0.37$, which is close to the transition point of $\Omega_{\mathrm{C}}^{\mathrm{L}} = 0.3753$. As shown in Fig.2a, the calculated Berry curvature for the bottom band is indeed localized in $\mathbf{k}$ space next to the transition points. Furthermore, the Berry curvature is an odd function under C_2 rotation, which is the consequence of T-symmetry. Accordingly, the Chern number of the band is always zero.

In comparison, our second calculation is for the Floquet Chern insulator example show in the right panel of Fig.3d in the main text, with a modulation frequency $\Omega a/2\pi c = 0.38$. As shown in Fig.2b, the Berry curvature is no longer an odd function in $\mathbf{k}$ space due to the absence of T-symmetry, while it is still localized near the transition point.

Supplementary Note 7: Super-cell setup for chiral edge state calculations

In this section, we present the super-cell setup in Fig. 4a in the main text, and explain how the edge and bulk states dispersion are calculated. First, we note that the external modulation applied in the domain-wall problem preserves translation symmetry in x direction, thus k_x is still a preserved quantity. Furthermore, by imposing periodic boundary condition along $1/2\hat{\mathbf{x}} + \sqrt{3}/2\hat{\mathbf{y}}$ direction, the trial solution of the Maxwell equation can be written in the form:

$$\begin{aligned}\Psi_{k_x}(t) &= e^{ik_x x - i\varepsilon t} \sum_{j,m,k_y} c_{j,m,k_y} \left|j(k_x, k_y)\right\rangle \frac{e^{ik_y y}}{\sqrt{N}} e^{im\Omega t} \\ &= e^{ik_x x - i\varepsilon t} \sum_{j,m,k_y} c_{j,m,k_y} \left|j, m, k_y\right\rangle\end{aligned} \tag{24}$$

where $k_y = (\frac{n}{N}\frac{4\pi}{a} - k_x)/\sqrt{3}$, N is the number of unit cells contained in the super-cell and n is an integer runs from 0 to $N-1$. It is easy to show that the basis states satisfy the orthonomal condition $\langle j', m', k'_y| B_0 |j, m, k_y\rangle = \delta_{j',j}\delta_{m',m}\delta_{k'_y,k_y}$. Substitution of the trial solution back into (1) and multiply $\langle j', m', k'_y|$ from the left, we obtain similar result as (3) with increased basis dimension:

$$\begin{aligned}&\sum_{j,m,k_y} c_{j,m,k_y}[(\omega_{j,k_y} + m\Omega)\delta_{j',j}\delta_{m',m}\delta_{k'_y,k_y} + m'\Omega V_{j'k'_y,jk_y}\delta_{m',m-1} + m'\Omega V^{\dagger}_{j'k'_y,jk_y}\delta_{m',m+1}] \\ &= \varepsilon \sum_{j,m,k_y} c_{j,m,k_y}(\delta_{j',j}\delta_{k'_y,k_y}\delta_{m',m} + V_{j'k'_y,jk_y}\delta_{m',m-1} + V^{\dagger}_{j'k'_y,jk_y}\delta_{m',m+1})\end{aligned} \tag{25}$$

where $V_{j'k'_y,jk_y} = \langle j', k'_y| V |j, k_y\rangle$ is the interacting matrix element that couples the orthogonal basis states:

$$V_{j'k'_y,jk_y} = \frac{1}{N}\sum_{n=0}^{N-1}\int_{\text{unit cell}} d^2\mathbf{r} u^{\star}_{j',k'_y} V u_{j,k_y} e^{i(k_y - k'_y)(y + na\sqrt{3}/2)} \tag{26}$$

where u_{j,k_y} is the periodic function of the electric fields. If the super-cell is subject to uniform external modulation, namely V is the same for each unit cell, there is no interface along y direction

and the interacting matrix element reads:

$$\begin{aligned} V_{j'k_y',jk_y} &= \frac{1}{N}\int_{\text{unit cell}} d^2\mathbf{r} u^{\star}_{j',k_y'} V u_{j,k_y} e^{i(k_y-k_y')y} \sum_{n=0}^{N-1} e^{i(k_y-k_y')na\sqrt{3}/2} \\ &= \int_{\text{unit cell}} d^2\mathbf{r} u^{\star}_{j',k_y} V u_{j,k_y} \delta_{k_y',k_y} \end{aligned} \tag{27}$$

On the other hand, when the super-cell consists of two regions (range from 0 to s and $s+1$ to $N-1$, respectively) being modulated with distinct polarization (V_1 and V_2), as the domain-wall problem considered in the max text, contributions from the two regions must be taken into account accordingly,

$$\begin{aligned} V_{j'k_y',jk_y} &= \frac{1}{N}\int_{\text{unit cell}} d^2\mathbf{r} u^{\star}_{j',k_y'} V_1 u_{j,k_y} e^{i(k_y-k_y')y} \sum_{n=0}^{s} e^{i(k_y-k_y')na\sqrt{3}/2} \\ &+ \frac{1}{N}\int_{\text{unit cell}} d^2\mathbf{r} u^{\star}_{j',k_y'} V_2 u_{j,k_y} e^{i(k_y-k_y')y} \sum_{n=s+1}^{N-1} e^{i(k_y-k_y')na\sqrt{3}/2} \end{aligned} \tag{28}$$

Therefore, the corresponding eigenvalue ε and eigenstate $\{c_{j,m,k_y}\}^{\mathrm{T}}$ for each k_x can be obtained by diagonalizing the Floquet Hamiltonian (25) in a similar way as we solve for the Floquet bulk spectrum in Supplementary Note 1.

Supplementary Note 8: Estimation of Floquet band gap size

In this section, we analyze the Floquet band gap size that can possibly be achieved with realistic nonlinear materials like $LiNbO_3$. From (9), it is found the induced Floquet gap size (normalized to $\sqrt{\omega_1\omega_2}$) by an external monochromatic drive is $\sim |V_{21}|$, which characterizes the coupling strength of the two modes mediated by the second order nonlinear process. For example,

when we consider the coupling between a TM ($|1\rangle$) and a TE mode ($|2\rangle$), it reads

$$
\begin{aligned}
|V_{21}| &= \int d^2\mathbf{r}\mathbf{E}^{\star}_{\mathrm{TE}}\bar{\bar{\epsilon}}_{\mathrm{nl}}\mathbf{E}_{\mathrm{TM}} \\
&= \frac{\epsilon_{xz}}{\epsilon_{\mathrm{l}}}\int d^2\mathbf{r}\epsilon_{\mathrm{l}}E^{\star}_{x,\mathrm{TE}}E_{z,\mathrm{TM}} + \frac{\epsilon_{yz}}{\epsilon_{\mathrm{l}}}\int d^2\mathbf{r}\epsilon_{\mathrm{l}}E^{\star}_{y,\mathrm{TE}}E_{z,\mathrm{TM}}
\end{aligned}
\tag{29}
$$

where the integral is performed over the area filled with nonlinear materials and $\epsilon_{xz}, \epsilon_{yz}$ terms are the effective permittivity induced by external drive. Limited by the material damage threshold, the maximal value of $\epsilon_{xz,yz}/\epsilon_{\mathrm{l}}$ for $LiNbO_3$ is estimated to be $\sim 5\times10^{-3}$. On the other hand, the modal overlap integral depends on the choice of bands and may varies from 10^{-2} to 1. As a result, the normalized Floquet gap size is typically on the order of $5\times10^{-5} \sim 5\times10^{-3}$.

To clearly show the Floquet gap in Fig. 3 in the main text, we assume large effective permittivity for both elliptically and linearly polarized drive: $(\epsilon_{xz}, \epsilon_{yz}) = (2, -1.08 + 1.9i)$ and $(\epsilon_{xz}, \epsilon_{yz}) = (-0.26, 2)$, respectively. The resulted gap size is $\sim 10^{-2}$. Therefore, we expect to obtain a gap size of $\sim 10^{-4}$ when the driving field strength is reduced by 100 times (4 GV·m^{-1}), which is below the material damage threshold. Similarly, for the chiral state dispersion calculation, we also assume large effective permittivity for the normal insulators: $(\epsilon_{xz}, \epsilon_{yz}) = (3, 1)$, which support a frequency gap aligned with the gap of the Chern insulator.

Supplementary References

1. Rudner, M. S., Lindner, N. H., Berg, E. & Levin, M. Anomalous edge states and the bulk-edge correspondence for periodically driven two-dimensional systems. *Physical Review X* **3**, 031005 (2013).

2. Boyd, R. W. *Nonlinear optics* (Elsevier, 2003).

3. Bach, F., Mero, M., Chou, M.-H. & Petrov, V. Laser induced damage studies of linbo 3 using 1030-nm, ultrashort pulses at 10-1000 khz. *Optical Materials Express* **7**, 240–252 (2017).

4. Shen, H., Zhen, B. & Fu, L. Topological band theory for non-hermitian hamiltonians. *Physical review letters* **120**, 146402 (2018).

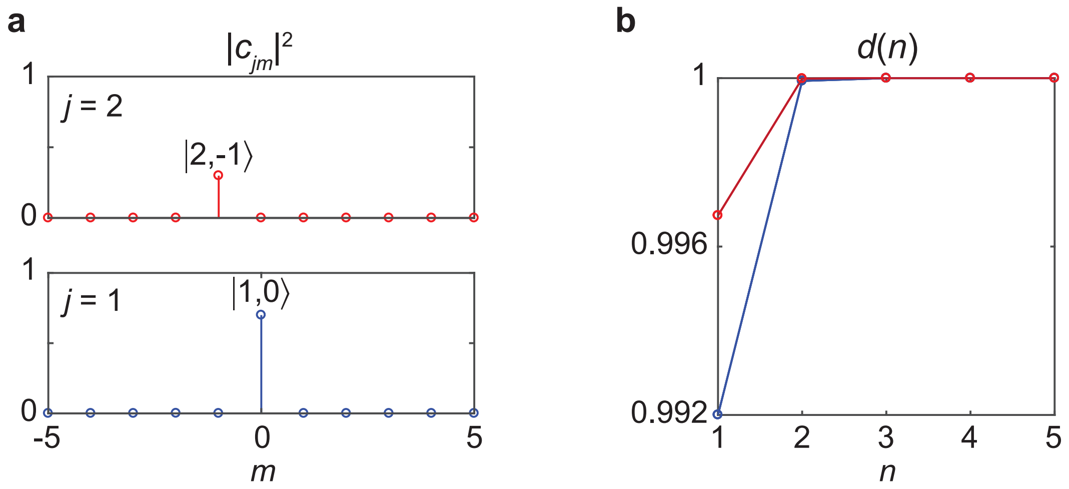

Supplementary Figure 1: | **The convergence of Floquet eigenstates calculation with truncated Floquet basis. a,** The localization of c_{jm} in m for a specific Floquet state (the bottom band at the Γ point in Fig.3d of the main text). **b,** The convergence of $d(n)$ for the two Floquet bands in Fig.3d of the main text.

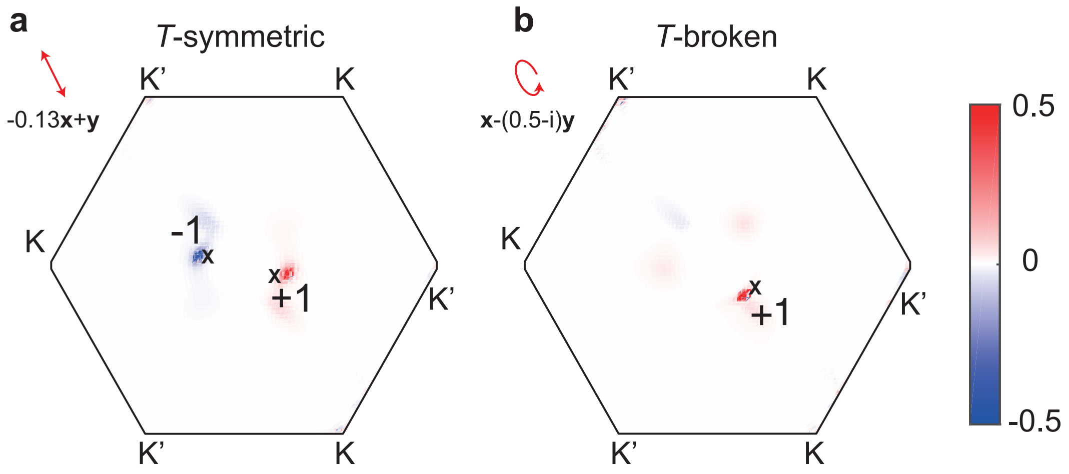

Supplementary Figure 2: | **Berry curvature distributions of Floquet bands with and without time-reversal symmetry. a,** Under linearly polarized drive, the calculated Berry curvature is an odd function of $\mathbf{k}$ and is localized near the two Weyl points (cross) due to the presence of time-reversal symmetry. **b,** In contrast, when the photonic crystal is driven by elliptically polarized light, as the case shown in Fig.3d of the main text, $\mathcal{B}_{\mathbf{k}}$ is no longer an odd function of $\mathbf{k}$, while it is still localized near the phase transition point (cross).
